# A SiPM-Enabled Portable Delayed Fluorescence Photon Counting Device: Climatic Plant Stress Biosensing

**DOI:** 10.3390/bios12100817

**Published:** 2022-10-02

**Authors:** William J. Pietro, Ozzy Mermut

**Affiliations:** 1 Department of Chemistry, York University, 4700 Keele St., Toronto, ON M3J 1P3, Canada; 2 Department of Physics and Astronomy, York University, 4700 Keele St., Toronto, ON M3J 1P3, Canada

**Keywords:** delayed fluorescence lifetime, in-field plant stress biosensor, vegetation climate stress analyzer, miniaturized photon counting, SiPM weak emission detection, drone plant sensor device

## Abstract

A portable and sensitive time-resolved biosensor for capturing very low intensity light emission is a promising avenue to study plant delayed fluorescence. These weak emissions provide insight on plant health and can be useful in plant science as well as in the development of accurate feedback indicators for plant growth and yield in applications of agricultural crop cultivation. A field-based delayed fluorescence device is also desirable to enable monitoring of plant stress response to climate change. Among basic techniques for the detection of rapidly fluctuating low intensity light is photon counting. Despite its vast utility, photon counting techniques often relying on photomultiplier tube (PMT) technology, having restricted use in agricultural and environment measurements of plant stress outside of the laboratory setting, mainly due to the prohibitive cost of the equipment, high voltage nature, and the complexity of its operation. However, recent development of the new generation solid-state silicon photomultiplier (SiPM) single photon avalanche diode array has enabled the availability of high quantum efficiency, easy-to-operate, compact, photon counting systems which are not constrained to sophisticated laboratories, and are accessible owing to their low-cost. In this contribution, we have conceived, fabricated and validated a novel SiPM-based photon counting device with integrated plug-and-play excitation LED, all housed inside a miniaturized sample chamber to record weak delayed fluorescence lifetime response from plant leaves subjected to varying temperature condition and drought stress. Findings from our device show that delayed fluorescence reports on the inactivation to the plant’s photosystem II function in response to unfavorable acute environmental heat and cold shock stress as well as chronic water deprivation. Results from our proof-of-concept miniaturized prototype demonstrate a new, simple and effective photon counting instrument is achieved, one which can be deployed in-field to rapidly and minimally invasively assess plant physiological growth and health based on rapid, ultra-weak delayed fluorescence measurements directly from a plant leaf.

## 1. Introduction

Global warming threatens to compromise our plant-based resources, the significance and effect to which it is yet unknown. The health, growth and production of healthy plants and consumer crops is intimately linked to temperatures and weather extremes [1], atmospheric greenhouse gases [2], salinity [3], soil nutrition [4]—all of which affect plant development and its sustainable production [5]. The increasing danger of climatological extremes, from floods to droughts related to surface temperature changes, are of concern and are widely believed to not only affect plant growth and photosynthesis [1,6] but also produce broader impact to the terrestrial vegetation ecosystems [7]. The consequences can have numerous detrimental effects to our food chain. The complex interactions between plant physiology and environmental shifts are not well understood nor its downstream impacts on plant resource availability in the face of climate change. Thus, there is an emerging need for biosensor technologies which are designed to diagnose plant health in situ within their ever-evolving environment [8].

An opportune way to investigate vegetation health is through a fundamental weak light emission process from plants known as “delayed fluorescence” which occurs in all photosynthetic species [9]. Although photosystem II (PSII) is primarily utilized for light photosynthesis, a small fraction of absorbed light energy from photoexcited chlorophyll may be reverted with charge recombination in the electron transport chain leading to P680 excitation, the chlorophyll reaction center of PSII [10]. This results in emission of photons at much longer timescales on the order of milliseconds to even hours compared to typical “prompt fluorescence” (with pico to nano second emissions) [11]. While the photosynthetic reactions and mechanisms associated with delayed fluorescence are highly complex and species-dependent within the photosynthetic apparatus of PSII [12], it is suggested that the kinetic behavior of this weak emission can be used to gauge plant physiological state [13,14] as a function of environment conditions. For example, water status [15], chlorophyll concentration [16], heavy metal pollution [17], pH and temperature stress [18,19,20], nutrient level and herbicide [21,22], have been reported to influence delayed fluorescence behavior making it an attractive, accessible and label-free biomarker to assess plant photosynthetic activity and monitor its health.

Compared to steady state measures vulnerable to optical fluctuations, time-resolved approaches to delayed fluorescence allow discrimination against light scattering, reflection, autofluorescence, and extraneous prompt fluorescence. To capture lifetimes of very weak delayed fluorescence from plants [23], accounting for a mere ~0.03% of the total emission [24], highly sensitive photodetectors must be employed, generally involving cost prohibitive, laboratory grade equipment such as a photon counting photomultiplier tube (PMT), a detector on the order of thousands of dollars. Recently however, silicon photomultiplier (SiPM) technology has enabled miniaturized detectors and photon counting to become an accessible at a fraction of the price, only a few hundred dollars. Similar to a PMT, a SiPM provides high-gain and a fast response in detecting radiation. Unlike bulky and fragile PMTs, the compact and rugged SiPM is driven with a much lower voltage and is more robust—amenable to operating in versitile external environments [25,26]. A silicon photomultiplier offers the high quantum efficiency advantage of solid-state semiconductor photomultiplier, with the capability of multipixel photon counting [27]. A single chip SiPM consists of an array of microcell avalanche photodiodes, each of which individually operate in single photon counting Geiger-mode conducive to sensitive, time-resolved detection even in the presence of magnetic fields. These properties, along with the small footprint of an SiPM detector have recently found new uses as a building block in a variety of biophotonics sensing devices from sophisticated medical imaging instruments [28] to compact, remotely deployable bio-chemiluminescence devices [29]. While instruments for capturing delayed fluorescence in laboratory environments exist [30,31,32,33,34], to the best of our knowledge, a portable, low-cost SiPM-based device with smart photon counting algorithm—affording supreme sensitivity for in-field biosensing of delayed fluorescence has not yet been reported to assess plant condition.

The aim of this work is to conceive a design, develop and characterize a novel, SiPM-integrated delayed fluorescence photon counting apparatus, hereon referred to as “York DF Photon Counting Device”, and demonstrate proof-of-concept function for measurement of plant-response to heat, chill and drought environmental stressors. Reciprocally, we propose that plant delayed fluorescence behavior, acquired from an extension of this remotely deployable technology, may be a viable biomarker to probe climatic changes based on a miniaturized implementation in a drone-based embodiment.

## 2. Materials and Methods

### 2.1. Plant Samples and Stress Exposure

**Figure 1:**
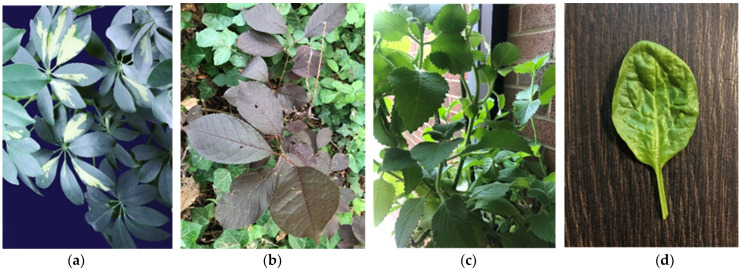
From left to right, the plants tested are (**a**) *Schefflera arboricola*, (**b**) *Prunus virginiana*, (**c**) *Coleus amboinicus*, and (**d**) *Spinacia oleracea*.

Delayed fluorescence was studied in the leaves of three healthy living plants gathered from the gardens of York University: (a) Dwarf Umbrella Tree (*Schefflera arboricola*), a common flowering house plant popular in North America, (b) Common Chokecherry (*Prunus Virginiana*), (c) Cuban Oregano (*Coleus amboinicus*), and (d) Spinach (*Spinacia oleracea*) purchased from Costco. Several leaves from each species (shown in Figure 1) were used in measurements, with five signal averaged measurements per species. Multiple heat and chill stress experiments were conducted with readily available and well-studied *Spinacia oleracea* to ensure similar trends in delayed fluorescence were observed. All photon counting studies were performed within one hour of cutting the leaf from the plant with the exception of the spinach leaf. All leaves were secured in the sample chamber with non-fluorescent tape and subjected to total darkness for 30 min for dark adaptation prior to delayed fluorescence measurements. All blank measurements were done with the lid of the sample chamber securely closed and including the non-fluorescent tape.

Using our newly constructed York DF Photon Counting Device, acute cold shock was studied for the outdoor plants for proof-of-concept delayed fluorescence biosensing. The delayed fluorescence lifetimes of the plant leaves were first measured under ambient conditions (21 °C), then subsequently placed in a refrigerator (3 °C), followed by a freezer (−17 °C) for 15 min prior to assessment of chill resistance.

As with cold-shock, we similarly exposed the plant leaves to acute heat stress and examined the time-dependent delayed fluorescence behavior with our photon counting SiPM biosensor upon heating a leaf from ambient conditions overtop a light-tight thermally regulated hotplate at 40 °C and 50 °C for 15 min. After both heat and chill shock stressors, the leaf exposed to these extreme temperatures was returned to 21 °C ambient conditions to examine delayed fluorescence recovery. All experiments were conducted in a stable, thermostatted environment at 21 °C.

Plants differ greatly in their ability to withstand drought conditions. Thus, we also investigated the effects of extended drought over time with the *S. arboricola* house plant. The delayed fluorescence of an *S. arboricola* cutting was monitored over 12 weeks of water deprivation. The cutting was continuously subjected to 12-h light and dark cycles. As with drought conditions, overwatering (flood stress) of the waterlogged soil for 3 days was also measured by way of the delayed fluorescence traces from our biosensor.

### 2.2. Design and Implementation of SiPM Miniaturized Photon Counting Modules and Device

**Figure 2:**
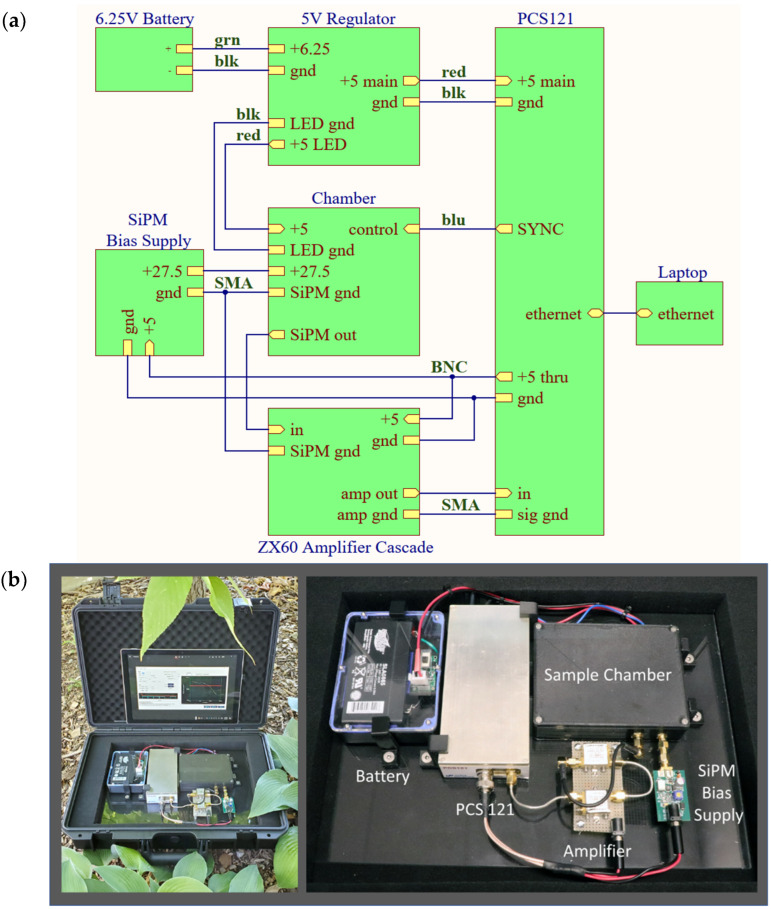
(**a**) Block diagram showing interconnections of modules (top). (**b**) Setup of the York DF Photon Counting Device having total footprint 30 cm × 23 cm inside a portable case. The system consists of the PCS121 High-Speed Pulse Counter, the SiPM amplifier, 27.5 V bias supply, lead-acid battery and voltage regulator, the delayed fluorescence sample chamber and a tablet user interface.

The block diagram and photograph of our system are shown in Figure 2. The device’s footprint is 30 cm × 23 cm. The system uses an ON Semiconductor model 10035 silicon photomultiplier (SiPM). This is a 1 mm^2^, blue-enhanced SiPM with 504 35 μm SPAD microcells and a gain of 3 × 10^6^ electrons per photon. ON Semiconductor SiPMs incorporate a “fast output”, a capacitively coupled output taken directly off each SPAD’s quench resistor, resulting in sub-nanosecond spikes on the order of a few hundred microvolts. In our system, the spikes from the fast output are amplified by two cascaded Mini-Circuits ZX60-43-S+ gigahertz amplifiers, with a summed gain of 40 db. The amplified spikes are sent to a United Nanophase PCS121 High-Speed Pulse Counter.

Figure 2 elaborates on the PCS121 module as part of the York DF Photon Counting Device. The input of the PCS121 incorporates a software-controlled voltage comparator to set the detection threshold voltage. As the amplified voltage spikes were measured to be 50 mV, we set the detection threshold at 45 mV. To keep the instrument portable and battery-operated, the 27.5 V bias power for the SiPM was generated from the PCS121’s “5V though” output by a boost regulator built in-house. The PCS121 is connected via Ethernet to a laptop computer running Windows 10.

The entire system operates off a single 5 V power supply consisting of a 6.25 V 1.2 amp-hour lead-acid battery (Interstate Batteries model SLA0865) and an in-house built 5.0 V low-dropout linear voltage regulator. This small battery enables portable operation suitable for any in-field in situ analysis as seen in Figure 2.

**Figure 3:**
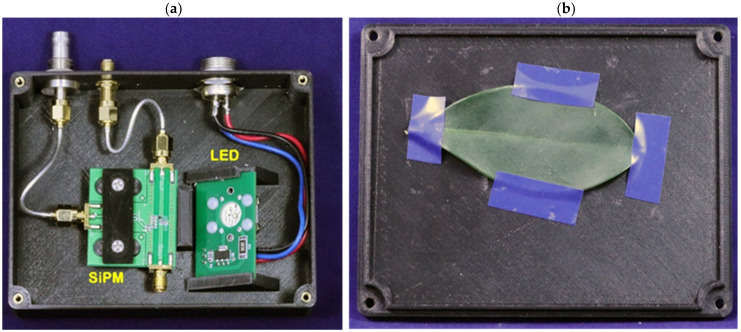
A photo of the inside of the chamber module having footprint: 9 cm × 12 cm. (**a**) Chamber base with the SiPM and interchangeable LED; (**b**) Chamber lid with the sample leaf adhered using non-fluorescent tape.

The sample chamber (9 cm × 12 cm) was constructed from black PLA using a 3D printer in two parts, the base and the lid. The base houses the SiPM module and the LED lamp assembly, as shown by the photograph in Figure 3. Non-fluorescent electrical tape was used to secure the leaf onto the inner side of the lid at a location that places it directly above the SiPM at a distance of 10 mm. The lamp assembly was secured at an angle directing light towards the leaf at a distance of 16 mm. Since delayed fluorescence emission from PSII is in the red and potential contaminant autofluorescence signals from the sample chamber would not contribute at the timescales measured herein, no emission filter was used.

The illumination assembly consists of a pulsed high-power LED (Lumileds Luxeon C series) driven at 1.0–1.2 W by an FZT603 power transistor operating as a saturated switch. Figure 3 illustrates our photon counting chamber is designed with a plug-and-play LED insert configuration wherein the Luxeon series LEDs are available in wavelengths ranging from 385 nm to 750 nm. This open architecture format allows a broad range of excitation wavelengths for delayed fluorescence studies, upon appropriate calibration of the SiPM spectral response. The FZT603 is a Darlington NPN that can be driven directly by the PCS121’s TTL SYNC output. Our experiment employed 530 nm light. This LED produced 141 mW of luminous power in a cone angle of 170°. At a distance of 16 mm, this produces a photon flux of 5.9 μmol m^−2^ s^−1^ impingent upon a leaf of typically 1.6 × 10^−3^ m^2^ surface area.

## 3. Results

### 3.1. Design Considerations for the SiPM

As the core engine of the photon counting delayed fluorescence apparatus, a deeper discussion of our resulting York DF Photon Counting Device and the algorithm for accurate photon counting is merited. A SiPM consists of an array of light-sensing elements called SPADs (single-photon avalanche diode). A SPAD is a special kind of diode that can remain in a metastable state when reverse-biased above its avalanche breakdown voltage. When a photon strikes the reverse-biased SPAD, an electron is promoted across the junction triggering avalanche breakdown associated with a sudden flood of electrons conducting through a resistor in series with the diode. The resulting voltage drop across the resistor, known as the “quench resistor”, opposes reverse-bias, cutting off avalanche. The SPAD then quickly resets back to its metastable state and is ready for another trigger. The voltage across the quench resistor appears as a sharp spike typically lasting a few hundred picoseconds and having an amplitude of a few hundred microvolts. Thus, by monitoring this voltage, the SPAD can detect a strike by a single photon. This is known as Geiger-mode operation.

An ideal SPAD should (a) only avalanche when struck by a photon, (b) always avalanche when struck by a photon, and (c) reset instantaneously after triggering. Unfortunately, real-life SPADs (a) do occasionally avalanche in total darkness, (b) have only a probability of avalanching when struck by a photon, and (c) require a finite amount of time to reset. Non-ideality (a) gives rise to the dark count rate, and it sets a lower limit to the sensitivity of an experiment. The consequence of non-ideality (b) limits the efficiency of photon detection and is aptly termed the photon detection efficiency (PDE, symbolized *γ*). Non-ideality (c), called the recovery time, sets an upper limit to the frequency at which individual photons can be discerned.

The SiPM used in our system is an array of 504 SPADs (*N_s_*), each integrated with its series quench resistor, connected in parallel to a single output. Thus, an avalanche from any SPAD in the array produces a voltage spike at the output. Each SPAD has a 31% (optimum) probability, *γ*, of avalanche upon being struck by a photon, has a broad spectral range of 300 to 950 nm (with varying wavelength sensitivity), a recovery time, *τ*, of 82 ns, and a dark count rate between 30 and 90 kHz at 25 °C (the dark count rate is exponentially dependent on temperature). In our 1 mm × 1 mm SiPM, the array spans a total active area, *A*, of 0.64 mm^2^.

The low detection limit of light intensity is set by the dark count rate. For a reliable photon count, the rate of photon-induced avalanches should be greater than about 10% of the dark count rate, or about 3 kHz of photon-induced avalanches. This corresponds to a photon flux, Φ, of 1.5 × 10^4^ photons s^−1^ mm^−2^.

The upper detection limit of light intensity is determined by the number of SPADs in the array. When a SPAD is hit more than once during its recovery time, it will only report the first photon. If the light intensity is high enough to continuously subject any given SPAD in the SiPM to multiple strikes within its recovery time, then the SPAD will be saturated and will report erroneous results. Here, we account for this by estimating the degree to which an SiPM is saturated as a function of light intensity based on probability that any given SPAD in the array will be hit twice within its recovery time by calculating,

(1) $$P_{2}={\left[1-{\left(\frac{N_{s}-1}{N_{s}}\right)}^{N_{\tau }}\right]}^{2}$$

(2) $$N_{\tau }=\Phi A\tau \gamma$$

where *N_τ_* is the number of avalanche-causing photons impingent on the array during its recovery time.

We determined that the probability reaches 10% at a photon flux of 1.2 × 10^10^ photons s^−1^ mm^−2^ and at this light intensity, 10% of the SPADs are saturated at any given time. Thus, at this light level, only 90% of the countable photons are actually being counted. Therefore, to ensure better than 90% accuracy, the light intensities used must be below this level. Nevertheless, our SiPM-based Photon Counting DF Device has a broad dynamic range of luminescence intensity detection of about six orders of magnitude.

### 3.2. Development of Software for SiPM Photon Counting Delayed Florescence

The PCS121 connects to the host computer via 10Base-T Ethernet using a UDP/IP protocol. United Nanophase provides Windows communication DLLs for their instruments to software developers. The PCS121 DLL contains C# callable methods to control every aspect of the PCS121. Thus, rather than use the PCS121 generic pulse counting software, we wrote our own software specific to a photon counting application. Writing our own software also enabled us to use the PCS121’s TTL SYNC output to control the LED. Hence, the PCS121 run by our software acts as a master controller for the entire delayed fluorescence experimental sequence.

**Figure 4:**
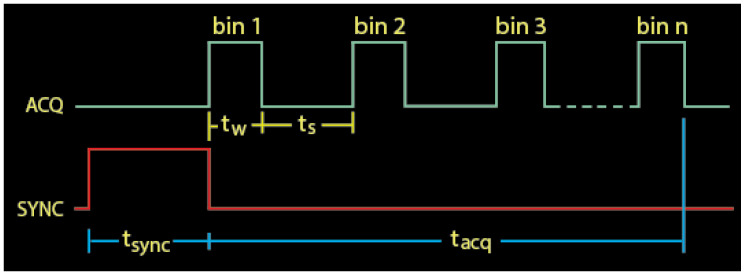
Timing sequence diagram for a delayed fluorescence experimental sequence.

The experimental sequence, shown in Figure 4, begins by setting the PCS121 SYNC output high for some number of seconds specified by *t*_sync_. This turns on the LED. Following *t*_sync_, the LED is switched off by clearing the SYNC output, and data acquisition starts immediately. The PCS121 slices time into a series of up to 250 discrete bins, each holding a 13-bit number representing the number of avalanches that occurred during its time slice.

Each bin is sequentially “opened” for duration *t_w_*, called the bin width, during which photons are counted. The time between successive bins is called the bin space, *t_s_*. The bin width can be set anywhere between 800 ns and 4 ms, the bin space can be set between 1 μs and 32.7 ms or set to zero, and the SYNC pulse can be set between 2 μs and 8.35 s. The total acquisition time, *t_acq_*, is

(3) $$t_{acq}=n_{bins}\left(t_{w}+t_{s}\right)-t_{s}$$

A number of scans are sequentially taken and averaged together for noise reduction. In our experiments, we averaged 5 scans. In these delayed fluorescence photon counting experiments, the 530 nm LED was activated for 10 s, then the acquisition scan initiated immediately after the LED was switched off. The timing parameters were set for a bin width of 500 μs and a bin space of 1 ms, for a total acquisition time of 374 ms to fill 250 bins, and a total scan time of 10.4 s.

It is important to note that the SiPM Geiger-mode time response is sub-nanosecond. For the purposes of experiments here, the time resolution of our acquisition system developed was 1.0 μs. This microsecond timescale limit is appropriate because unlike prompt fluorescence (on the timescale of nanoseconds), delayed fluorescence decay in plants is typically millisecond to seconds.

#### 3.2.1. Estimation of Higher-Order Counts

When a photon-induced avalanche occurs in a single SPAD, a current spike of magnitude *i_a_* is produced which is then amplified and recorded by the associated circuitry. The SiPM, together with its associated circuitry, however, has a finite response time, *τ*. If two SPADs avalanche within time period *τ* of each other, only one spike will be produced, albeit double in magnitude (2*i_a_*). If *m* SPADs avalanche within period *τ*, one spike of magnitude *mi_a_* is produced. We refer to this as an *m*-level spike producing an avalanche of degree *m*.

Conventional avalanche counting systems associated with the SiPMs do not simply count avalanche spikes, but simultaneously analyze their magnitudes for multiple current levels to determine how many single, double, triple, quadruple, etc., hits occurred in each time bin. This requirement markedly increases the cost and complexity of the counting circuitry. In addition, directly counting occurrences of multiple strikes is complicated by the phenomenon of crosstalk between SPADs, a potential source of inaccuracy when SiPMs are operated in Geiger mode.

In our software, we introduce an alternate method for estimating contributions of higher-order spikes requiring only a rudimentary count without discernment of magnitude, and uncomplicated by crosstalk. The method is based on determining the probability that exactly *m* avalanches (*m* = 1, 2, 3, …) occur within the response time of the SiPM and associated circuitry, *τ* (*τ* << *t_w_*) in a given bin time window of width *t_w_*, during which time *N_a_* avalanches occurred.

We divide the time bin of width *t_w_* into a number of time “buckets” each of width τ. The number of buckets, *N_b_*, is,

(4) $$N_{b}=\frac{t_{w}}{\tau }$$

After randomly distributing *N_a_* avalanches throughout *t_w_*, we calculate how many buckets will contain exactly one avalanche, or exactly two or exactly *m* avalanches. The solution is given by the binomial distribution,

(5) $$p_{m}=\left(\begin{array}{c} N_{a}\\ m \end{array}\right)P^{m}{\left(1-P\right)}^{N_{a}-m}$$

where *p_m_* is the probability of finding a bucket containing *m* avalanches, *P* is the probability that an avalanche will occur in any given time bucket,

(6) $$P=\frac{1}{N_{b}}$$

and $\left(\begin{array}{c} N_{a}\\ m \end{array}\right)$ is the binomial coefficient. Thus,

(7) $$p_{m}=\left(\begin{array}{c} N_{a}\\ m \end{array}\right){\left(\frac{1}{N_{b}}\right)}^{m}{\left(\frac{N_{b}-1}{N_{b}}\right)}^{N_{a}-m}$$

Statistically, the number of buckets, *n_m_*, containing exactly *m* avalanches is, *n_m_ =* p*_m_*N*_b_*. These numbers relate directly to the observed spike count, *C_obs_*, by,

(8) $$C_{obs}=\overset{N_{a}}{\underset{m=1}{\sum }}n_{m}$$

Importantly, *C_obs_* is the total number of current spikes counted by the avalanche counter independent of their level. This is a single count of all spikes without discernment of magnitude. As an alternate interpretation of Equation (8), *C_obs_* is the number of buckets that are not empty. Thus,

(9) $$C_{obs}=N_{b}\left(1-p_{0}\right)=N_{b}-N_{b}{\left(\frac{N_{b}-1}{N_{b}}\right)}^{N_{a}}$$

Solving for *N_a_*,

(10) $$N_{a}=\ln\left(\frac{N_{b}-C_{obs}}{N_{b}}\right){\left[\ln\left(\frac{N_{b}-1}{N_{b}}\right)\right]}^{-1}$$

Therefore, solving Equation (10) provides the total number of avalanches, including all *m*-level avalanches, given only a simple measurement of *C_obs_*, greatly simplifying data acquisition.

The number of photons, *N*, impingent on the SiPM during time *t_w_* is *N = N_a_/γ*, where *γ* is the SiPM’s photon detection efficiency (a function of wavelength, overvoltage, active area, and the number of SPADs in the array). With *N* known, the photon flux, Φ, and luminous power, *P*_Φ_, can also be determined,

(11) $$\Phi =\frac{N}{t_{w}A}$$

(12) $$P_{\Phi }=\frac{h\Phi c}{\lambda }$$

where *A* is the active area of the SiPM (0.64 mm^2^ in our case). The PCS121 communication DLL contains routines for exporting CSV files, which our software uses to export time vs. either photon count, *P*_Φ_ (W mm^−2^), or Φ (photons mm^−2^ s^−1^).

#### 3.2.2. Crosstalk

All silicon photomultipliers exhibit the phenomenon of crosstalk. When a SPAD avalanches, it emits infrared photons, as many as a hundred per avalanche. A fraction of these photons can reflect off the SiPM’s front window and trigger a secondary avalanche of another SPADs in the array, thereby mimicking multiphoton coincidences. Fortunately, these secondary photons mostly affect only between four and eight nearest neighbors to the primary SPAD, relatively few photons are reflected off the window, and the photon detection efficiency at infrared wavelengths is very low. Nevertheless, crosstalk can present accuracy problems in photon counting systems. Crosstalk only manifests in our device as higher-level spikes, and therefore, our detection is not substantially affected by crosstalk. Insensitivity to crosstalk affords another advantage in our York DF Photon Counting Device described herein.

#### 3.2.3. Measurement of Higher-Level Avalanches

**Figure 5:**
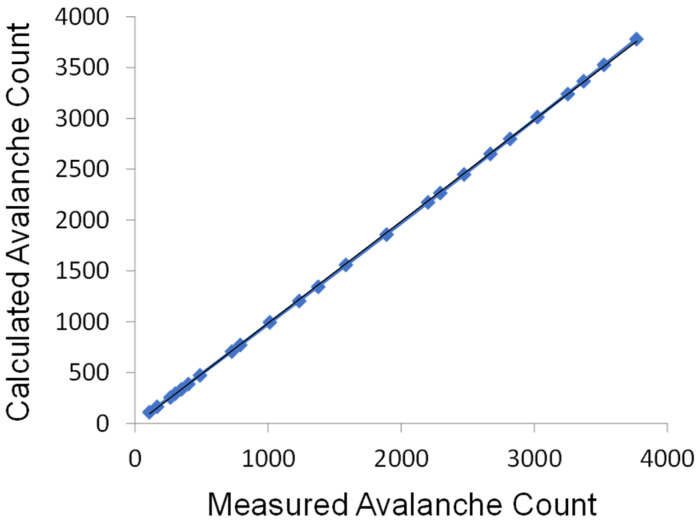
Comparison of avalanche count calculated by Equation (10) with observed avalanche count by discrete measurement of *n_m_*, 1 ≤ *n_m_* ≤ 4. All instrumental parameters were experimentally determined and are presented in Supplementary Materials (Table S1). The comparison line has a slope of 1.000 (ideal fit = 1.000) and an R^2^ value of 0.9999.

As the PCS121 allows us to set voltage thresholds for detection, we ascertained the accuracy of our algorithm for estimating higher-order avalanche counts by comparison to direct measurement obtained by the method described in the Supplementary Materials. The result is presented in Figure 5 showing a near perfect linear behavior of the calculated avalanche count vs. the measured avalanche count, with R^2^ value of 0.9999. These results indicate an excellent correlation, implying it is not necessary to measure the voltage of each avalanche event and instead it can be very accurately counted.

### 3.3. SiPM Photon Counting in Measuring Delayed Fluorescence

**Figure 6:**
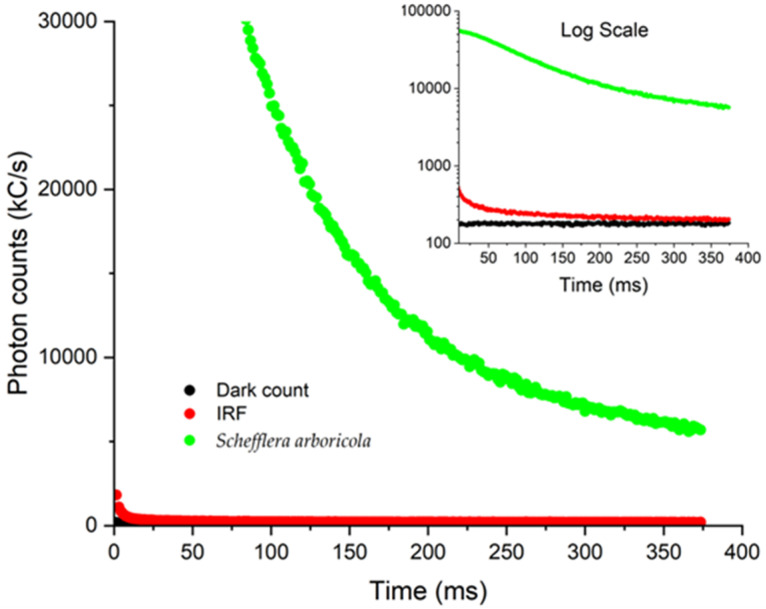
Photon vs. time curves plotted for the dark counts (black), the background blank including the IRF (red), and a sample delayed fluorescence response curve from *Schefflera arboricola* leaf (green). Inset is the log plot.

Figure 6 shows the photon counts vs. time graphs recorded for the dark counts, background sample chamber, and representative delayed fluorescence decay from the leaf of the common houseplant *Schefflera arbicola*. Our device software GUI enables visualization of decay graphs for immediate qualitative assessment.

The dark count rate was measured to be 41 kHz. Although dark count caused by spontaneous avalanches in the SiPM does not result from photon strikes, it does not interfere with the experiments as it was well below the luminescent count rate from all leaves tested ~15 MHz (using a photon detection efficiency of 0.31 for the SiPM used).

The background control experiment, in which the leaf was not present in the chamber, shows the initial expected peak from the instrument response function (IRF) representing a convolution of the LED excitation with the SiPM detector electronics, amplification circuitry, as well as any luminescence signal from the background chamber. The IRF is acquired just when the excitation is shut off (immediately after SYNC period) and the $t_{acq}$ begins. The response time of the SiPM amplifier was measured to be 22 ns. Our results show that the IRF is dominated by the lag decay from the LED, space-charge capacitance due to oversaturation of the SiPM, and the excitation transistor’s response time, collectively observed to be 1.6 ms. Given timescale of milliseconds to seconds typical for delayed fluorescence, the IRF does not present a significant contribution nor experimental challenge in acquiring valid delayed fluorescence photon count decay data from the leaves. Fits for delayed fluorescence lifetime were obtained starting at 3 ms removed from the IRF. In measuring the background, we also observed the presence of a slight luminescence from the chamber contributing to the IRF and not much higher than the dark count rate, that decayed rapidly on a timescale of a few milliseconds. This is likely due to a trace amount of either phosphorescence or autofluorescence in the LED, and it does not interfere with the delayed fluorescence measurements, as it is of very low intensity and exhibits rapid decay.

### 3.4. Photon Counting Biosensing

The complex biophysics of delayed fluorescence in PSII is well beyond the scope of this paper. However, details of the fascinating processes are covered in excellent reviews of the subject [10,11,12]. Here, our goal was to verify functionality and validate our York DF Photon Counting Device in the context of demonstrating a plant delayed fluorescence biosensor. Although delayed fluorescence can be multi-mechanistic and highly species dependent, it is generally known that delayed fluorescence decay behavior can be described and fit to a sum of exponentials relaxations [35,36],

(13) $$DF\left(t\right)=\overset{n}{\underset{i=1}{\sum }}A_{i}e^{-\frac{t}{\tau_{i}}}$$

where DF is the delayed fluorescence intensity, at time t once the LED excitation light is switched off. The coefficient $A_{i}$ represents the amplitude and $\tau_{i}$ is the characteristic relaxation lifetime of the ith component. In our plant studies, we observed apparent bi-exponential decay kinetics in all cases. With the fit data, we determined the lifetime values $\tau_{1}$ and $\tau_{2}$ to quantify relative changes in delayed fluorescence in response to environmental stressors. We benchmarked changes to the delayed fluorescence comparing to the fresh, healthy leaf under ambient conditions (21 °C).

The intra-sample variance observed in the absolute photon counts is attributed to the changing positioning of the leaf when placed back into the chamber upon heating or chilling, producing variant absorption, and fluorescence along the optical path reaching the SiPM. Hence, a key advantage of using time-resolved photon counting over absolute photon counts (steady state delayed fluorescence intensity), is that lifetime provides an absolute measure of the intrinsic fluorescence behavior independent of such extrinsic factors. It is a more optically robust modality for stress characterization compared to relative intensity measurements, being less sensitive to parasitic light reflections and sample placement. Measuring decay kinetics with the York DF Photon Counting Device is therefore more reliable for in-field plant stress determination where the sample cannot be well-controlled, as in a laboratory setting, and requires less management of spurious stray light interference from the plant or its environment.

#### 3.4.1. Photon Counting Biosensing: Impact of Acute Heat Stress

**Figure 7:**
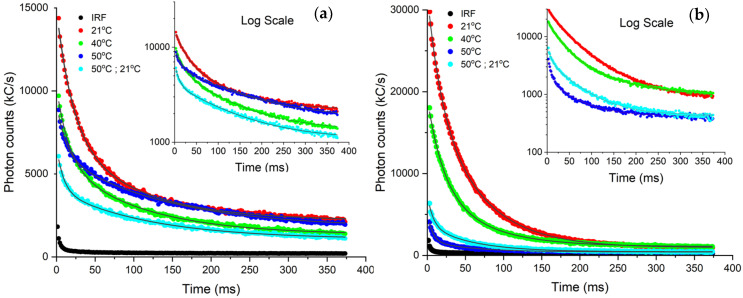
Photon lifetime curves acquired from our York DF Photon Counting Device for heat stressed: (**a**) *Prunus virginiana* and (**b**) *Coleus amboinicus.* The plants were subjected to temperatures 21 °C (red), 40 °C (green), 50 °C (blue), 50 °C returned to ambient (cyan). Black line indicates the instrument response function.

Figure 7 presents the photon count vs. time curves of two species leaves, *Prunus virginiana* and *Coleus amboinicus* which were subjected to acute heat exposure. The exponential decay fits are summarized in Table 1. It was both observed and determined from the decay lifetimes that *Prunus virginiana* delayed fluorescence behavior changed as the leaf was heat shocked from ambient temperature (21 °C) to 40 °C, and finally 50 °C. After the leaves where stressed, a measurement of potential recovery of delayed fluorescence was tested termed “50 °C, then ambient”. Upon extreme heat exposure, the $\tau_{1}$ for *Prunus virginiana* showed a trend of steady decrease in its values from 20.8 ms to 8.8 ms, while the $\tau_{2}$ tended to increase relative to ambient 21 °C. Overall, the response of delayed fluorescence appeared to be reduced with heat shock.

Interesting, the opposite behavior was observed for the *Coleus amboinicus* species in its $\tau_{1}$ value, increasing from 17.2 ms to 21.1 ms upon raising the temperature from 21 °C to 40 °C, with subtle changes to $\tau_{2}$. While one still observes some small, delayed fluorescence at 50 °C, it was clear however that at this elevated temperature, the PSII was impacted, and delayed fluorescence diminished. The lifetime fit errors on the order of 2–5% imply that the changes in delayed fluorescence characteristics measured in both *Prunus virginiana* and *Coleus amboinicus* species are significant. These heat shock results are consistent with reports [37] suggesting that excess heat, as small as 5 °C, can interfere with the cytoskeleton, cellular and organelle structure, protein transcription, membrane function [38], and metabolic functions [39]. Plants sense environmental temperature and signaling cascades are altered with influx of ions such as Ca^2+^, and inhibiting oxygen evolving complex linked to PSII. These various factors can cause damage to the photosystem and its repair mechanism [40]. Our heat stress findings of the leaves studied with our SiPM-based photon counting device are corroborated with previous kinetic studies observing perturbations in delayed fluorescence of pea leaves incubated at 20–50 °C where the results are believed to arise from a decrease of electron transport rate in PSII at high temperatures [41]. Temperature stress affects photosynthetic activity also by way of damage to thylakoid membranes [42]. For example, soybean plants exhibit a decrease in delayed fluorescence intensity with increasing temperature [36]. Not surprisingly, we found similarly measuring with our device that the delayed fluorescence heat stress tolerance was highly species-dependent [37,43,44].

#### 3.4.2. Photon Counting Biosensing: Impact of Acute Chill Stress

**Figure 8:**
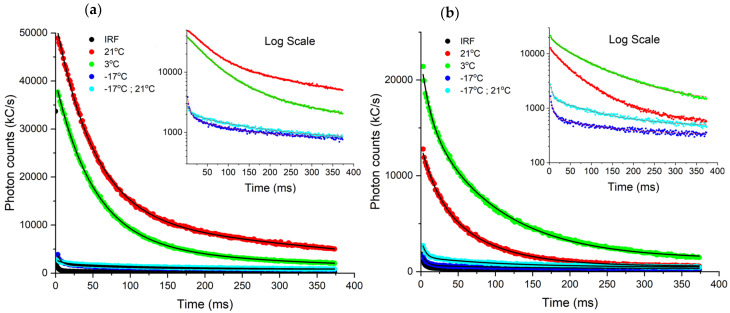
Photon lifetime curves acquired from York DF Photon Counting Device for chill stressed: (**a**) *Spinacia oleracea* and (**b**) *Coleus amboinicus.* The plants were subjected to temperatures 21 °C (red), 3 °C (green), −17 °C (blue), −17 °C returned to ambient (cyan). Black line indicates the instrument response function.

Studies of various vegetation including tomato and wheat leaves, have shown that delayed fluorescence induction kinetics change radically with temperature decrease [45]. Along with a diminished yield, this delayed fluorescence decay response has been closely correlated with alterations of membrane integrity and its energy state [45]. Since chill stress has previously been investigated in *Spinacia oleracea* by means of delayed fluorescence, we next decided to interrogate it and the *Coleus amboinicus*, (Cuban oregano) species. The Cuban oregano plant thrives indoors and in conditions ranging from 15.6–21.1 °C, but is not well-adapted for colder climates. Results of the delayed fluorescence response upon inducing chill stress (21–17 °C) to the two plants are shown in Figure 8, with lifetime fits synopsized in Table 2.

Data acquired from our PCS121 Photon Counting DF Device, indicated that both plant species exhibit drastic diminishment of delayed fluorescence after 15 min of cold shock exposure to freezing temperature. This was evidenced by in the lifetime reduction in $\tau_{1}$, changing an order of magnitude from 52.4 ms to 5.3 ms for spinach when temperature dropped from 21 °C to −17 °C. Multiple chill stress experiments, conducted with readily available spinach, consistently revealed the same trend. Results acquired from our York DF Photon Counting Device corroborate previous findings of delayed fluorescence measured in market spinach chloroplasts exhibiting second order kinetics [46] with diminished yield upon exposure to freezing conditions at −10 °C—behavior suggestive of formation and dissipation of PSII oxidized products [45]. *Coleus amboinicus* similarly showed loss of most of its delayed fluorescence signal along with $\tau_{1}$ lifetime weakening from 22.0 ms to 5.4 ms when ambient temperature was reduced to −17 °C.

For milder chill temperature exposure to 3 °C, the spinach leaf was more robust, where in fact, no major change to the $\tau_{1}$ was observed indicating a plant that preserves its photosynthesis activity in a refrigerated environment. The observed chill resistance of spinach at 3 °C may in part explain why this consumer product stays relatively healthy in a grocery store environment. Conversely, the more temperature-sensitive *Coleus amboinicus* showed nearly a factor two reduction in its $\tau_{1}$ lifetime, when exposed to 3 °C shock. Interestingly, both species show contributions from longer lived delayed fluorescence processes on the order of 100 ms, as determined by $\tau_{2}$. While $\tau_{2}$ trends towards shorten in lifetime with chill stress for spinach, it seems to increase under analogous conditions for *Coleus amboinicus*.

#### 3.4.3. Photon Counting Biosensing: Impact of Chronic Drought

**Figure 9:**
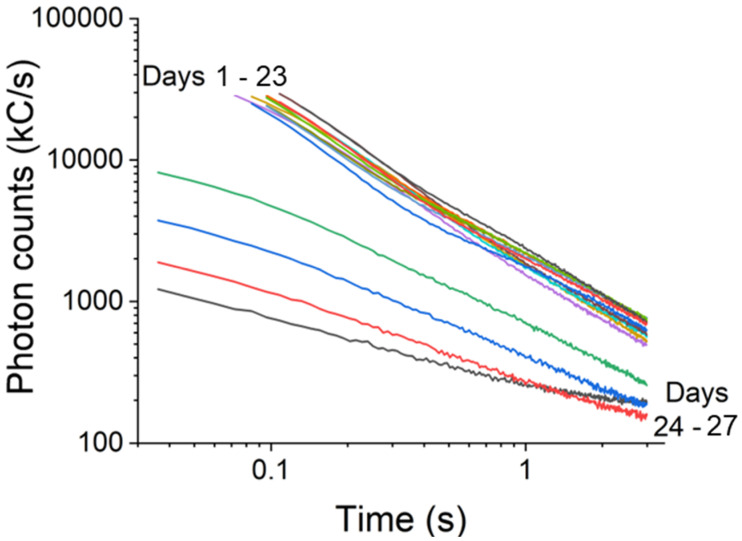
Biosensing chronic drought with York DF Photon Counting DF Device in *Schefflera arbicola* plant.

We finally examined chronic conditions of drought stress with the common house plant *Schefflera arbicola* under 12 weeks of water deprivation. The *S. arboricola* is highly robust against drought. In fact, one reason it is such a popular house plant is because it requires very little watering. Under the same instrument conditions, delayed fluorescence scans were taken at the same time every day 48 h apart. Results shown in Figure 9 display that for the first 24 days, the delayed fluorescence traces are nearly superimposable on one another. The delayed fluorescence intensity decreases very slightly with each day of water deprivation, and the fluorescence lifetimes remained mostly constant.

To compare extremes, the $\tau_{1}$ on day 1 increased from 47.8 ms to 82.7 ms on day 27, after which the droughted *S. arboricola* exhibited extremely diminished delayed fluorescence. On day 24, the leaves looked, by appearance, as healthy as it did on day 1, however, the measured delayed fluorescence suggested otherwise. On day 24 of drought, the delayed fluorescence curve exhibited a sudden, and remarkable, deviation, with its intensity dropping dramatically, the overall lifetime increasing, and the multi-process, bi-exponential behavior, becoming less pronounced. This pattern has been previously observed and explained as resulting from drought-induced impairment of the electron transport function in photosystem II [47,48], and reduction of chlorophyl content along with ultrastructural deformations of plastids [49]. After day 24, the delayed fluorescence from the leaf continued to rapidly deteriorate, yet, only after 26 days did the cutting begin to show visible signs of ill health. At that point, the delayed fluorescence intensity had been consistently reduced by a factor of about 24 and the multi-process structure vanished, coalescing into a simple exponential. After 50 days, delayed fluorescence was no longer detectable.

Conventional instructions for taking care of *S. arboricola* is to add water only when the leaves start wilting, and not add water if the soil is already moist. It is also commonly known that *S. arboricola* doesn’t fare well with chronic overwatering. Thus, as with drought, we tested the effects of overwatering (flood stress). We detected changes in the bi-exponential behavior in the time-resolved delayed fluorescence traces of flooded *S. arboricola*. Flooding similarly produced a marked loss of intensity as with drought, but interesting after two days in dry soil, the delayed fluorescence recovered back to normal intensity and shape.

## 4. Discussion

### 4.1. Photon Counting Biosensing: Design Considerations

We have demonstrated here a successful design and build of a proof-of-concept biosensor, SiPM-based York DF Photon Counting Device, showing promising results for capturing delayed fluorescence—an indicator for plant health under environmental stressors. Using our novel system, we exposed four plants to varying climatic conditions, and successfully measured plants’ responses to acute heat and chill shock as well as chronic drought stress by examining the modification to delayed fluorescence behavior. Our prototype reported on the status of the plant photosynthetic apparatus as a function of temperature shock, which varied according to the plant species and its corresponding temperature sensitivity, marked by observable changes to its lifetime. Whereas delayed fluorescence from plants have been restricted to acquisitions using laboratory grade equipment, employing costly, high voltage, large area photon counting detection systems, we have presented here a remotely deployable alternative—based on our SiPM photon counting approach—one which enables detection of ultra-weak delayed fluorescence emissions traditionally reserved to instrumentation utilizing PMT detectors. Results from our plant chill, heat, drought stress experiments reveal that this compact technology, having robustness and sensitivity imparted by time-resolved SiPM photon counting, is a suitable biosensor both to study delayed fluorescence as a scientific research tool, and to environmentally survey in situ photosynthetic biomarkers of vegetation without requiring application of exogenous contrast makers, complex laboratory sample processing, or sophisticated equipment.

Our study does show some limitations for capturing faster response time kinetics of delayed fluorescence of <1.5 ms, arising from the convolution of the excitation source response time and the instrument response function. This temporal limit of detection is dominated by the decay characteristics, namely fall time, of the LED. While the inexpensive SiPM used has a maximum response time of ~900 ps, we did observe a subtle slope in the baseline and an initial fast decay believed to be caused by intermittent SiPM saturation when exposed to extreme light intensities from the LED. When oversaturated, the avalanche electrons cannot flow out of the SiPM faster than they are created, the SiPM charges. Beyond approximately 100 million photons/s light intensity can affect the response time of the SiPM. We hypothesize that space charges are created within the SiPM that take about 100 μs to discharge, then an additional ~50 ms to fully recover from thermal effects from the LED, which can be seen in IRF baseline having a slight slope. It is important to note that this effect is a minor contribution, a fraction of the dark count in magnitude, and can be ignored for our measurements. Nevertheless, we suggest avoiding the oversaturation limits by incorporating a neutral density filter and scaling the intensity of the excitation LED so that delayed fluorescence kinetics can interrogate timescales of microseconds to minutes [50]. For the purposes of delayed fluorescence experiments here, the temporal limit of detection was more than adequate, and the incorporation of the neutral density filter was deemed unnecessary. However, broadening the dynamic range of delayed fluorescence timescales available would render a more comprehensive device allowing a variety plant physiological mechanisms and species to be interrogated [14]. While this device development study focused on delayed fluorescence on the millisecond timescale, SiPM SPAD arrays have a fundamental time resolution of 30 ps for photon counting [51]. This implies future designs are possible with simultaneous collection of both prompt fluorescence (100 picosecond to nanosecond timescales) and delayed fluorescence for enhanced plant stress monitoring capability, for example of relevance to analyzing ozone sensitivity, salinity, drought, and filtered UV exposure [52].

### 4.2. Future Embodiements: Delayed Fluorescence Biosensing on a Drone

**Figure 10:**
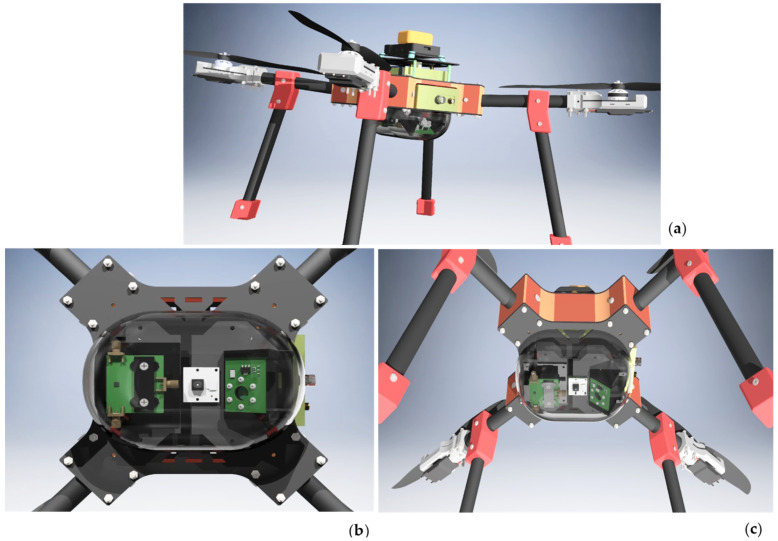
Conceptual design of a drone-based York DF Photon Counting Biosensor. (**a**) Top image shows the full view of the drone-integrated York DF Photon Counting Biosensor with PCS121 electronics, SiPM bias supply and amplifier integrated into the drone body; (**b**) Left image reveals the belly view of the optical elements of the device within dome, including the SiPM, target camera in the center, and excitation LED; (**c**) Right image displays a hovering drone bottom view. Credit: Biosensor integrated drone design by Haktan Yağmur [54].

Given its battery operation, light mass, and miniaturized design concept, one valuable implementation of the York DF Photon Counting Device would be in a remote, aerial biosensor platform [53]. As a future vision we propose a next step in prototyping a drone-based architecture. Longitudinal data acquisition of delayed fluorescence on a drone would economically enable unmanned, regular monitoring of forests and agricultural fields to analyze vegetation health over its growth stages. Data may also provide in situ feedback with periodic assessments correlated to weather events and climate extremes. Such a system could ascertain water drainage efficacy, examine crop health, map areas of soil stress or nutrient deficiency, gauge effects of spray fertilizers or pesticides, thereby delivering vital information to increase farming efficiency and yield. Protected lands, conservations, and forests could be surveyed with a drone-based configuration potentially identifying trends correlated to climate change. Figure 10 presents a possible architectural concept developed for a drone-integrated York DF Photon Counting Biosensor.

The design includes a transparent polycarbonate window encasing the SiPM photon counting module as well an excitation module to protect the optoelectronics from environmental elements as well as filter UV, IR light. The system operated at night, would minimize light below saturation and ensure Geiger mode photon counting by adding an adjustable iris to the SiPM. A high brightness white LED incorporates collimating and focusing optics for close range measurement, on the order of ~1 m above the plant. The SiPM detection module is constructed with a tunable delayed fluorescence spectral filter, such as solid state acousto-optic tunable filter, operating by varying RF power operated through PCS121 and providing the advantage of wide spectral range tuning with no moving parts. A PCB-level thermostat is easily integrated into the LED and SiPM module. SiPM detectors are well known to exhibit temperature dependence which affects gain, photon detection efficiency and optical crosstalk [55]. Although lifetime measurements are intrinsically less sensitive to these affects then delayed fluorescence intensity, it is vital to ensure system is thermostatted and a temperature calibration curve is established to accurately acquire lifetime data in outdoor environments. Recently, SiPMs have shown promising results even when operating without cooling, in advanced multiphoton imaging applications [56]. These studies suggests that despite a higher background dark count compared to a PMT, the SiPM can achieve a high SNR without the need to integrate a cooling module. An uncooled configuration provides advantage in weight and portability for drone-based operation of the SiPM-based photon counting delayed fluorescence biosensor. An ultra-low-cost signal multiplier would be built to lock-in [57] delayed fluorescence signal and remove interference from spurious light present even at nighttime.

Several remote sensing optical methods for arial farmland imaging have been reported using multi-spectral detection, thermal, LiDAR, and solar irradiance imaging which analyze photosynthetic activity from chlorophyll and are used to estimate of crop health. However, such approaches tend to employ costly optical instrumentation, or rely on satellite data. The Normalized Difference Vegetation Index (NDVI) often calculated from remote sensing equipment employed in broad geographical agriculture assessment, uses red-NIR spectral reflectance to determine chlorophyll. Such measurements, however, have shown limited efficacy in analyzing later stages of plant growth where chlorophyll concentration reaches saturation limits and can be sensitive to long-range atmospheric effects [58]. A low-cost drone architecture of our York DF Photon Counting Device may offer a complimentary optical solution to these existing surveillance toolboxes—one potentially circumventing some limitations of current long-range remoting sensing optical instrumentation, and one less sensitive to variability from steady-state measurements. We envision a time-resolved delayed fluorescence sensor on a drone may autonomously, and regularly provide functional feedback from large area vegetation and report on the impact of anomalous climatic variations.

## 5. Conclusions

Remotely deployable biosensing of plant health is of relevant current interest—from researching plant physiology to important technological applications related to understanding effects of greenhouse gas production, its impact on food and energy security, and evaluating environment sustainability. We have designed, developed and demonstrated here a novel, miniaturized SiPM-based York DF Photon Counting Device for in-field delayed fluorescence biosensing of photosynthetic activity in four different plants. This technology establishes a new paradigm for accessible delayed fluorescence biosensing—where conventional systems are price- and field- operation prohibitory. This portable, SiPM photon counting device was tested and validated by successfully monitoring perturbations to the plant photosystem II engine induced by exposure to heat, chill, and drought stress. We propose that a future prototype can be integrated into a drone configuration for continuous evaluation of large area agricultural and forestry lands to survey changes as a function of environmental conditions. Reciprocally, we suggest that such new photon counting device may serve in the study and development of new biomarkers to longitudinally assess impacts of climate change possibly measured by way of plant weak emissions.

## Figures and Tables

**Table 1:** Lifetime values of heat stressed *Prunus virginiana* and *Coleus amboinicus*.

*Prunus virginiana*			*Coleus amboinicus*		
Conditions (°C)	$\tau_{1}\;(\mathrm{ms})$	$\tau_{2}\;(\mathrm{ms})$	Conditions (°C)	$\tau_{1}\;(\mathrm{ms})$	$\tau_{2}\;(\mathrm{ms})$
21	20.8 ± 0.7	96.4 ± 4.0	21	17.2 ± 0.6	60.9 ± 0.7
40	15.9 ± 0.6	105.1 ± 2.8	40	21.1 ± 0.7	67.1 ± 2.1
50	11.6 ± 0.7	135.3 ± 3.6	50	6.5 ± 0.2	55.1 ± 1.6
50, return 21	8.8 ± 0.5	119.4 ± 2.9	50, return 21	7.2 ± 0.3	57.1 ± 1.70

**Table 2:** Lifetime values of chill stressed *Spinacia oleracea* and *Coleus amboinicus*.

*Spinacia oleracea*			*Coleus amboinicus*		
Conditions (°C)	$\tau_{1}\;(\mathrm{ms})$	$\tau_{2}\;(\mathrm{ms})$	Conditions (°C)	$\tau_{1}\;(\mathrm{ms})$	$\tau_{2}\;(\mathrm{ms})$
21	52.4 ± 0.3	- *	21	22.0 ± 1.3	70.8 ± 1.6
3.0	50.4. ± 0.7	243.6 * ± 52.7	3.0	12.6 ± 0.6	97.0 ± 1.1
−17	5.3 ± 0.3	100.9 ± 4.6	−17	5.4 ± 0.3	116.9 ± 7.9
−17, return 21	10.1 ± 1.0	156.2 ± 7.5	−17, return 21	4.9 ± 0.3	107.4 ± 3.4

* did not converge.

## Data Availability

Not applicable.

## Supplementary Materials

The following supporting information can be downloaded at: https://www.mdpi.com/article/10.3390/bios12100817/s1, Table S1: Experimental parameters for SiPM/amplifier/counter system; Table S2: Comparison of measured and calculated avalanche count in 1000 s time bin.
