# Supplementary material for: A SiPM-Enabled Portable Delayed Fluorescence Photon Counting Device: Climatic Plant Stress Biosensing
Source: Biosensors (Basel). 2022 Oct 2;12(10):817. doi: 10.3390/bios12100817 (PMC9599934; doi:10.3390/bios12100817)
Supplement: Supplementary file 1 [file biosensors-12-00817-s001.zip › biosensors-1928392-supplementary.pdf]

biosensors MDPI

Article

Supplementary Information:

A SiPM-Enabled Portable Delayed Fluorescence Photon Counting Device: Climatic Plant Stress Biosensing

William J. Pietro [1,†] and Ozzy Mermut [2,*,†]

[1] Department of Chemistry, York University, 4700 Keele St., Toronto, ON M3J 1P3, Canada
[2] Department of Physics and Astronomy, York University, 4700 Keele St., Toronto, ON M3J 1P3, Canada
* Correspondence: omermut@yorku.ca
† These authors contributed equally to this work.

Supplimentary Information for section 3.2.3. Measurement of Higher-Level Avalanches

Single and higher-level avalanche voltage spikes were measured with a 2 gigasample per second digital oscilloscope (LeCroy WaveAce 234). Single avalanches produce amplified voltage spikes of nominally 50 mV (post-amplifier) driving a 50 Ω transmission line with 50 Ω termination. Double avalanches were measured to be 100 mV, 150 mV for triple, and 190 mV for quadruple.

At the input of the PCS121 is a high-speed PECL analog comparator with a software-selectable precision reference voltage. Reference voltages of 45 mV, 90 mV, 140 mV, and 185 mV, were used to count single, double, triple, and quadruple avalanche events, respectively. Hence, we can directly measure the numbers of higher-order spikes for comparison with equation 5. The specifications of the SiPM and associated circuitry used in equation 5 are presented in Table S1.

A Würth Elektronik model 150120BS75000 blue LED emitting at 465 nm was used as the light source. Luminous intensity was varied between 0 and 290 mcd by regulating the current through the LED to a maximum of 20 mA. The SiPM was covered with a cap containing a 0.5 mm diameter pinhole. The path length from the LED to the SiPM was 26 mm.

Table S1. Experimental parameters for SiPM/amplifier/counter system.

Parameter	Symbol	Value
Active area	A	0.64 mm^2
SPAD recovery time	τ_r	82 ns
LED wavelength	λ	465 nm
Photon detection efficiency	γ	0.29 at 465 nm
Measured circuit response time	τ	22 ns
SiPM operating voltage	V	27.5 V
Measured dark count rate	C_d	55 kHz
Bin width	t_w	1000 μs

A count at level m, x_m, for an SiPM contains all spikes at level m and higher. For example, when measuring current spikes at level 1, the count, x_1, contains all level 1 and higher spikes. Count x_2 contains all level 2 and higher spikes, etc. Therefore, we generate a corrected set of experimental counts, d, such that $d_i = x_i - x_{i+1}$.

We measured up to level 4 current spikes, hence $d_4 = x_4$. The number of avalanches occurring in time window t_w is,

$$N_a(exp) = \sum_{m=1}^{4} md_m = \sum_{m=1}^{4} x_m \quad \text{(S1)}$$

The observed total number of spikes, C_{obs}, is simply x_1. Table S2 compares the avalanche count, N_a, by discrete measurement of x_i (i = 1-4) with the avalanche count estimated by Equation 10 in the manuscript.

Table S2. Comparison of measured and calculated avalanche count in 1000 µs time bin.

LED current (mA)	C_{obs}	N_a	
		**Measured (Eq. 6)**	**Calculated (Eq. 1 (SI))**
0.25	111	111	111.0
0.49	165	167	165.0
0.88	256	261	257.0
1.06	294	301	295.0
1.25	337	345	338.0
1.44	385	396	387.0
1.87	473	487	475.0
2.96	702	723	707.0
3.29	765	789	772.0
4.39	985	1017	996.0
5.48	1190	1231	1206.0
6.22	1326	1371	1346.0
7.37	1535	1588	1561.0
9.00	1820	1884	1857.0
10.86	2125	2203	2176.0
11.41	2210	2291	2266.0
12.51	2383	2472	2448.0
13.79	2575	2672	2651.0
14.77	2715	2818	2799.0
16.22	2915	3025	3013.0
17.70	3126	3245	3239.0
18.66	3244	3370	3366.0
19.86	3395	3526	3528.0
21.81	3627	3770	3780.0
